# Plant reproduction research in Latin America: Toward sustainable agriculture in a changing environment

**DOI:** 10.1002/pei3.10143

**Published:** 2024-05-17

**Authors:** Arnaud Ronceret, Pablo Bolaños‐Villegas

**Affiliations:** ^1^ Instituto de Biotecnología/Universidad Nacional Autónoma de México (UNAM) Cuernavaca Morelos Mexico; ^2^ Fabio Baudrit Agricultural Research Station University of Costa Rica Alajuela Costa Rica; ^3^ Lankester Botanical Garden University of Costa Rica Cartago Costa Rica; ^4^ Faculty of Food and Agricultural Sciences, Rodrigo Facio Campus, School of Agronomy University of Costa Rica San Jose Costa Rica

**Keywords:** biodiversity, innovation, Latin America, meiosis, plant embryo development, plant reproduction, pollen tube development, sustainable agriculture

## Abstract

Food production and food security depend on the ability of crops to cope with anthropogenic climate change and successfully produce seed. To guarantee food production well into the future, contemporary plant scientists in Latin America must carry out research on how plants respond to environmental stressors such as temperature, drought, and salinity. This review shows the opportunities to apply these results locally and abroad and points to the gaps that still exist in terms of reproductive processes with the purpose to better link research with translational work in plant breeding and biotechnology. Suggestions are put forth to address these gaps creatively in the face of chronic low investment in science with a focus on applicability.

## Graphical abstract

The sustainability of Latin American agriculture in the face of climate change may depend on advances on plant reproduction research performed by local groups.

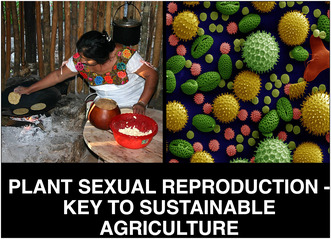

## 1 INTRODUCTION

Latin America is a heterogeneous ethno‐geographic region with a population of about seven hundred million that spans Mexico to Argentina and Chile (Belbin et al., 2018). Although it is not a single polity, it shares common cultural and social traits derived from European colonization about five hundred years ago (López et al. 2017). It is also the home of many of the world's most important crops owing to millennia of plant breeding by the cultures of Mexico, Central America, and the Andes (Gepts, 2010). Some of the crops are well known, such as maize (Guzzon et al., 2021), common bean (Rendón‐Anaya et al., 2017), potatoes (Fajardo‐Escoffié, 2022), tomatoes (Razifard et al., 2020), avocado (Rendón‐Anaya et al., 2019), papaya (Yue et al., 2022), cacao (Bolaños‐Villegas & Argüello‐Miranda, 2019), and vanilla (Watteyn et al., 2023). Others are less well known but still important in terms of food security and cultural identity for local communities. These crops include amaranth (Olmos et al., 2022), quinoa (Isobe et al., 2018), and cassava (Bolaños‐Villegas & Argüello‐Miranda, 2019).

Enhancing agricultural productivity is key to raising living standards; however, human activities during the last century and a half have raised global temperatures by more than 1°C above preindustrial values (Ortiz‐Bobea et al., 2021). In crops such as maize, high temperatures above 40°C may induce photosynthetic stress and impact the flowering and grain filling stages, thus causing sterility (Ureta et al., 2020). Thus, for instance in Brazil, rising temperatures may reduce yields in maize and soybean by 29%–36% (Zilli et al., 2020), while in Latin America, the whole agricultural output may decrease by 25% (Ortiz‐Bobea et al., 2021). Other expected negative effects of climate change on agriculture are as follows: (1) an increase in the frequency of extreme weather events such as extended drought and intensive rainfall, (2) changes in ultraviolet irradiance, (3) desertification and erosion, and (4) salinization brought about by changes in irrigation and leaching (Corwin, 2021; Ortiz et al., 2021).

This review will cover the state‐of‐the‐art in plant reproduction research in Latin America with the purpose of identifying opportunities for agricultural innovation and collaboration to foster adaption to climate change, while also addressing potential gaps that may require rethinking of current approaches to agriculture and agricultural research, including the way academia interacts with the rest of society.

## 2 SNAPSHOT OF CONTEMPORARY LATIN AMERICAN RESEARCH IN PLANT REPRODUCTION

How does Latin America fare in terms of research into plant reproduction when compared with the rest of the world? In contrasting bodies of literature, there is an imbalance between the output of Latin America, Organization for Economic Cooperation and Development (OECD) countries (as a proxy for the Collective West), and the rest of the developing world, including the rising scientific powerhouse China. For instance, for 2022, results from using the *Shape of Science* visualization tool Scimago (Hassan‐Montero et al., 2014) suggested that for the fields of agricultural and biological sciences, biochemistry, genetics and molecular biology, and plant science, the total combined output across Scientific Citation Index (SCI) journals (Q1 to Q4) of an unspecified number of articles was 3998. From these, most authors hailed from OECD countries (*n* = 3689 journals, or 92%), followed by the Middle East (*n* = 2884, or 72%), Latin America (*n* = 2793, or 69%), China (*n* = 2734, or 68%), all of Africa (*n* = 2615, or 65%), and India (*n* = 2415, or 60%). This is a rough comparison because of lack of a proper search item for “plant reproduction”(instead, we used a combination of proxy terms associated with agriculture and molecular biology); because a few Latin American countries are OECD members themselves (https://www.oecd.org/about/document/ratification‐oecd‐convention.htm); because of the common practice that Latin American and doctoral students from the Global South may publish as part of established laboratories in North America and Europe; and because of the high likelihood that a substantial portion of the scientific literature in developing countries is only available locally.

A quick analysis of the international literature also hints that for contemporary plant reproduction research, there is a clear focus on the study of processes that mediate genetic recombination (Cheng et al., 2022; Wang et al., 2023), pollen tube attraction (Mizuta et al., 2023), and embryo development (Karami et al., 2023) in mature plant models such as *Arabidopsis thaliana*, which is not a crop, or rice and maize, which double as plant models and crops. In these three cases, the work does not focus on field observations or phylogenetic studies but rather on basic research that may be translated into enhanced crop yields, which is a pressing need considering population growth and climate change (Lenaerts et al., 2019). For instance, the work of French researcher Raphäel Mercier in meiosis has paved the way for clonal rice in which meiotic recombination is abrogated (the Mitosis instead Meiosis phenotype, or *MiMe*). Then, ectopic expression of the sperm identity factor *BABYBOOM1* (Arabidopsis gene ID *At5g17430*) allows for autonomous embryo development (Stokstad, 2023). This type of work allows for the indefinite fixation of maximal heterosis in rice, a crop consumed by billions of people across every continent (Stokstad, 2023). Another example is the work of R.K. Dawe and colleagues in the United States that led to the breeding of haploid maize lines (Dawe et al. 2023). This work cleverly used mutant alleles for the centromeric histone *CENH3* (Arabidopsis gene ID *At1g01370*; maize gene ID *ZmB84.06G235300*), and male lines that express the *cenh3* allele contribute chromosomes via the sperm nuclei. These chromosomes then fuse with the haploid egg cell and the diploid central cell and disintegrate during cell division (Dawe et al. 2023).

## 3 MEIOSIS

A line of work of great interest is the establishment of crossovers between homologous chromosomes during plant meiosis. Meiotic crossovers are physical links between homologous chromosomes that are formed due to the exchange of parental sequences (Rafiei & Ronceret, 2023). Their distribution and number along chromosomes are not random, but in general at least one obligate crossover will be formed between each pair of chromosomes to guarantee proper segregation during metaphase I (Girard et al., 2023). Two major pathways exist for meiotic crossover formation: interfering class I and non‐interfering class II. Class I crossovers represent most crossovers (80% in Arabidopsis) and interfere with each other's formation, whereas those of class II are in the minority; the two classes do not show mutual interference (Lian et al., 2022). Regulation of the class I pathway is under control of factors ZIP4/SPO22/PH1 (a tetratricopeptide repeat *TPR*‐like superfamily protein), MER3 (a DNA helicase), HEI10 (a RING finger‐containing protein), PTD (a DNA ligase‐like protein), SHOC1/ZIP2 (an XPF endonuclease‐like protein), and the resolvases MSH4, MSH5, and MLH1 (Rafiei & Ronceret, 2023). However, formation of class II crossovers is regulated by two pathways: MUS81 and Fanconi Anemia Complementation Group D2 (FANCD2) pathways (Rafiei & Ronceret, 2023). The endonuclease MUS81 (GEN1 in rice) targets recombination intermediates such as D‐loops and Holliday Junctions to promote crossover formation (10%–15%), and the FANCD2 pathway promotes crossover formation at a rate of 5% (Kurzbauer et al., 2018; Rafiei & Ronceret, 2023).

Notably, the team of Ronceret et al. at the National Autonomous University of Mexico (UNAM) (see Table 1) in collaboration with Rachel Wang at Academia Sinica in Taipei has provided evidence that in the maize *spo11‐1* mutants (Ku et al., 2020), the induction of double‐strand breaks, crossover formation, and the very organization of the axial elements (the backbone) and chromosome loops is all compromised. In eukaryotes, the topoisomerase SPO11 (Arabidopsis *At3g13170*, maize *Zm00001d013262*) regulates the formation of double‐strand breaks in DNA, and in these maize mutant meiocytes, the formation of double‐strand breaks is severely impaired, as shown by TUNEL assay. Consequently, homologous chromosomes are unable to establish crossover sites and few bivalents are formed (Ku et al., 2020). Homologous chromosomes also become long and curly by zygotene, a phenotype also observed by Mathilde Grelon in the Arabidopsis *zyp1‐1* mutants, in which differences in crossover frequency between male and female gametes (heterochiasmy) are abolished (Durand et al., 2022). In the case of maize, the actual mechanism underlying these changes is not known (Ku et al., 2020); however, changes in crossover distribution or formation rates clearly occur when the anti‐helicase FANCM is mutated, as seen in in Arabidopsis (Crismani et al., 2012) and lettuce (Li et al., 2021). One‐third of the global maize cultivation area is in tropical areas of low‐ and middle‐income countries, for instance Latin America, East Africa, Sub‐Saharan Africa, and South‐East Asia, all of which are drought‐prone areas (Prasanna et al., 2022). In these countries, past production gains were a result of an increase in area, not yield (Prasanna et al., 2022). Thus, novel breeding techniques based on manipulation of recombination in crops are top priority to boost yield (Epstein et al., 2023; Prasanna et al., 2022).

**TABLE 1:** Selected leading Latin American institutions in plant reproduction research as of 2023–2024.

Name of institution	Location	Areas of expertise, issue addressed	Researchers showcased	Impact on food security and agricultural adaptation to climate change	Requirements for implementation
The National Autonomous University of Mexico, Plant Molecular Biology Department, Institute of Biotechnology, Cuernavaca, México	Instituto de Biotecnología/UNAM, Avenida Universidad #2001, Colonia Chamilpa C.P. 62210, Cuernavaca, Estado de Morelos, México, (52777) 329 16 00	Meiosis is a cellular process that allows for the transfer of traits during conventional plant breeding.	Arnaud Ronceret, arnaud.ronceret@ibt.unam.mx	Enhanced meiosis may allow for quick development of new conventional non‐transgenic lines that are stress and drought‐tolerant in crops such as maize, amaranth, and quinoa; thus, local landraces are used as allele reservoirs that can be tapped indefinitely for either the breeding of elite lines or for communal/traditional agriculture.	May require investment on rural seed banks for the conservation of agricultural diversity and may require an upgrade of local gene sequencing facilities. May require the training of extension workers able to perform conventional plant breeding
The National Autonomous University of Mexico, Plant Molecular Biology Department, Institute of Biotechnology, Cuernavaca, México	Instituto de Biotecnología /UNAM, Avenida Universidad #2001, Colonia Chamilpa C.P. 62210, Cuernavaca, Estado de Morelos, México, (52777) 3291600	Seed Developmental biology. Salt intrusion in coastal areas and high salinity in large intensive farms affect seed germination. Use of plants models such as Arabidopsis thaliana for the identification of genes that confer resistance to salinity is the first step in the breeding of salt‐tolerant crops.	Alejandra A. Covarrubias, alejandra.covarrubias@ibt.unam.mx	Food security is threatened by rising sea levels. High sea levels cause sea water intrusion into reservoirs across coastal areas. Also, high evaporation rates in the tropics lead to salt deposition in agricultural soils. Usually, seeds do not germinate under high salinity. Crops bred to tolerate salinity may be the key to stabilizing production by resorting to marginal soils.	Requires the molecular characterization of local crops to identify homologs of Arabidopsis salinity‐ tolerance genes. Requires local plant breeding by either the government or the private sector.
Botany Institute of the Northeast (IBONE‐CONICET‐UNNE), Department of Agricultural Sciences, National University of the Northeast, City of Corrientes, Corrientes Province, Argentina	Facultad de Ciencias Agrarias, Universidad Nacional del Nordeste, (FCA‐UNNE), 3400, Corrientes, Argentina	Sexual reproduction in *Paspalum* grasses. *Paspalum* grasses are stress‐tolerant grasses of great agricultural importance in Argentina, such as the *P. notatum* tetraploid cultivar “Argentine” (PI 148996) released in the 1950s, and are still sown as a pasture and utility turf.	Eric J. Martínez, eric@agr.unne.edu.ar	The efficiency of *Paspalum* breeding pipelines would increase by boosting the number of apomictic hybrids produced, and better selecting for highly self‐incompatible hybrids.	May require high initial investments of facilities for plant molecular biology and sequencing, but the social return due to high pasture yield will compensate.
Londrina State University, Laboratory of Cytogenetics and Plant Diversity, City of Londrina, Paraná State Brazil	Laboratório de Citogenética e Diversidade Vegetal (LCDV), Universidade Estadual de Londrina (UEL), Londrina, PR 86057–970, Brasil	Meiosis and pollen development in holocentric grasses. Holocentric weeds from *Cyperaceae* are very adaptable organisms that cause losses in crops such as maize and rice. A better understanding of their reproductive biology may facilitate agricultural management.	André Luís Laforga Vanzela, andrevanzela@uel.br	Food security in the tropics is threatened by the extremely adaptable and resilient weed *Cyperus rotundus*. Plants from the *Cyperaceae* family are extremely resilient and can only be controlled through aggressive herbicide use. Climate change and heat reduce the efficiency of herbicides. Thus, a better understanding of reproduction in *Cyperaceae* may facilitate control.	May require high investments on cell biology facilities but may also be of interest to herbicide manufacturing companies.
The National Autonomous University of Mexico, Department of Biochemistry, Mexico City	Facultad de Química, Universidad Nacional Autónoma de México, Circuito Exterior S/N, Coyoacán, Cd. Universitaria, 04510 Ciudad de México, CDMX, México, (55) 56223512	Seed developmental biology in maize calli for tissue culture. Tissue culture of maize is required for the rescue of embryos in crosses between lines that are far apart genetically. Tissue culture is stressing and activates jumping genes known as transposable elements (TEs). By understanding what genes silence TEs, it might be possible to choose lines with high expression of these repressors and thus enhance calli survival.	Tzvetanka Dimitrova Dinkova, cesy@unam.mx	Maize is native to the Americas as is extremely rich in genetic diversity. However, genes of interest for drought, stress, and pest tolerance may reside in species or lines that are genetically isolated. Rescue of embryos in the lab may allow for the quick transmission of genes from thousands of lines, landraces, and species stored at the seed bank of CIMMYT in Mexico State.	Requires sophisticated equipment for molecular biology and aseptic laboratories for tissue culture. May also require the training of faculty and students at key agricultural universities throughout Latin America.
Formerly a student of Natalia Pabón‐Mora, at the Institute of Biology, University of Antioquia, Medellín, Colombia, now at Università degli Studi di Milano (Italy)	Via Celoria 26, Università degli Studi di Milano Milano, Italy, 20,133	Seed developmental biology under differences in genome size. Parental species or lines may harbor differences in genome size; this often causes collapse of the seed endosperm, a reservoir of energy for the embryo, and the largest source of seed protein and carbohydrates for human beings.	Cecilia Zumajo‐Cardona, cecilia.zumajo@unimi.it; Ignacio Ezquer, juan.ezquer@unimi.it	Identification of genes that rescue endosperm development in interspecific crosses, such as Arabidopsis *TRANSPARENT TESTA 8* (At4g09820), may allow for gene and allele introgression from wild relatives.	The use of model plants such as Arabidopsis thaliana for the functional characterization of genes is not common in Latin America. The exchange of seed and protocols must be encouraged. Local seed banks should be set up at universities.
Mexican National Laboratory of Genomics for Biodiversity (LANGEBIO) at the Center for Advanced Research (CINVESTAV), Irapuato, Mexico	Langebio, Unidad de Genómica Avanzada, CINVESTAV‐IPN, Libramiento Norte León Km 9.6, 36,821 Irapuato, Guanajuato, México +524,621,663,000	Seed developmental biology and genomics. Enhanced embryo development in polyploid lines may allow for the breeding of local wheat varieties.	Stewart Gillmor, stewart.gillmor@cinvestav.mx	Wheat does not grow well on acidic tropical soils, such as the Brazilian Cerrado. However, development of new drought‐tolerant lines in Mexico and Argentina may allow for local harvests and may strengthen food security.	Wheat is a staple grown in South America. Training of extension workers on wheat breeding by the Mexican CIMMYT institute on may be required to boost yield. Upgrade of local gene sequencing facilities may also be required.
Institute of Biological Research, Mar del Plata University, Argentina	Instituto de Investigaciones Biológicas, Universidad Nacional de Mar del Plata, Consejo Nacional de Investigaciones Científicas y Técnicas (CONICET), 7600 Mar del Plata, Argentina +54,223,472–4143	Seed developmental biology and seed abortion. Seed abortion is often caused by defects in the development of the ovule or in defects during pollen tube guidance. Identification of genes that regulate these two processes may lead to enhanced rates of fertilization and seed set or to intentional abortion.	Gabriela C. Pagnussat, gpagnussat@mdp.edu.ar	Identification of Arabidopsis regulators of mitochondrial electron transfer such as the adrenodoxins (ADX1, At4g05450; ADX2, At4g21090 and ADXR, At4g32360)suggest that it is possible to reduce seed abortion below 10 percent. Also, it may be possible to do the opposite and induce sterility to better control pollen reception in high‐yield double and triple hybrids, as done in rice.	Molecular characterization of genes that control seed set is best done in model plants such as Arabidopsis thaliana. Latin American universities must encourage its use as part of breeding programs or even as a teaching tool for genetics.
Laboratory of Functional Genomics, Institute of Biological Sciences, University of Talca, Chile	Universidad de Talca, Talca 3,460,000, Región del Maule, Chile +56 712,200,277	Pollen developmental biology. Pollen tube elongation is key for the fertilization of plant ovules and for seed production.	Simón Ruiz‐Lara, sruiz@utalca.cl, Carolina Puentes, apuentes@utalca.cl Carlos R. Figueroa, cfigueroa@utalca.cl	Elongation of the pollen tube is critical for fertilization of ovules and the formation of seed for human consumption. Climate change imposes stress during fertilization because of heat or because of drought. A better understanding of molecular regulators of pollen tube elongation and ovule development such as *AtZAT4* (At2g45120) may stabilize seed set under suboptimal cultivation conditions.	Crops have life cycles that are very long. Thus, it is hard to isolate gene mutants that illustrate gene function. Use of model plant Arabidopsis thaliana is a cost‐effective to study plant developmental processes of local relevance before moving onto crops. Latin American universities must encourage the adoption of plant models and the free exchange of seed for basic and applied agricultural research.
Millennium Nucleus for the Development of Super Adaptable Plants (MN‐SAP), Chile & Institute of Research in Genetic Engineering and Molecular Biology, National Scientific and Technological Research Council of Argentina, Argentina	Instituto de Investigaciones en Ingeniería Genética y Biología Molecular, Dr. Héctor Torres 6 (INGEBI‐CONICET), Vuelta de Obligado 2490, Buenos Aires, C1428ADN CABA, Argentina +54 114,783–2871	Pollen developmental biology and pollen tube elongation	José M. Estévez, jose.estevez@unab.cl & Jorge P. Muschietti, prometeo@dna.uba.ar	Pollen tube elongation is a process required for efficient fertilization of ovules. Quick and uniform elongation rates facilitate seed development under environmentally challenging circumstances. Their work in Arabidopsis thaliana has shown that quick elongation depends on the activity of receptor kinases, of the hydroxyproline‐rich glycoprotein (HRGP) superfamily. Identification of gene homologs in South American crops may boost harvests under drought or heat stress, a common situation now situation in Argentina, the South of Chile and in the Amazon Basin of Brazil.	The small model plant Arabidopsis thaliana offers a quick life cycle, and easy cytological procedures, specially to study pollen tube elongation. Its use is recommended as a cost‐effective approach to the identification of genes that regulate sexual reproduction. Latin American universities and research centers depend on seed banks in the Global North to purchase mutant seed. The free exchange of Arabidopsis mutant seed should be encouraged as part of extended breeding initiatives.
Department of Biology, University of Sao Paulo, Brazil	Departamento de Biologia, Faculdade de Filosofia, Ciências e Letras de Ribeirão Preto, Universidade de São Paulo, Ribeirão Preto 14,040–901, São Paulo, Brasil +55(16) 3315–3670	Pollen developmental biology and seed formation	Maria Helena S. Goldman, mgoldman@ffclrp.usp.br	Seed formation depends on effective pollen tube elongation and pollen tube guidance. Identification of genes that regulate this process (such as pectin acetylesterases) may be attempted on Nicotiana tabacum, a model plant system that allows transient gene transformation via transfection. *Nicotiana* is easy to grow and allows detailed subcellular characterization of proteins that cannot be performed on most crops due to long life cycles and recalcitrance to transfection.	High seed set is key for high productivity in the tropics. The identification of key genes in *Nicotiana tabacum*, such as NtPAE1, may allow for the breeding of drought‐resistant crops not commonly grown in lush South America such as olives.

*Note*: Information collected and collated from publications and institutional websites.

Unfortunately, despite the enormous importance of meiosis for plant breeding, biology majors in Latin America may not have a clear grasp of the process, as shown by the study of Rodríguez Gil et al. (2019) at the UNAM (Mexico), in which students were not able to complete a simple diagram. Also, the overall proportion of GDP allocated to science in Latin America is a serious limiting factor for any type of research (Bolaños‐Villegas et al., 2020). However, there are multiple examples of applied research in the field of meiosis, as shown by the cytogenetic work in Mexico of Ruvalcaba‐Ruiz and Rodríguez‐Garay (2002) in blue agave, in which structural rearrangements reduce pollen fertility; the work of Usandizaga et al. (2020) in Argentina with tetraploid and hexaploid lines of forage grass *Acroceras macrum* Stap.; and the work of Sattler et al. (2016) in Brazil with coffee, specifically with the allotriploid (2*n* = 3*x* = 33) interspecific hybrid *Híbrido de Timor*, a great source of resistance to pathogens that was resurrected by tissue culture from only four plants.

Of outstanding relevance is the work of Verena Reutemann and Eric J. Martínez at Universidad Nacional del Nordeste in Argentina (see Table 1). They have characterized gene flow in diploid (2*n* = 2*x* = 20) species of tropical *Paspalum* grass that may either outcross or self‐mate (Reutemann et al., 2023). These are agriculturally important turfgrasses highly tolerant to salinity and drought (Wu et al., 2020). Contrary to what was expected by Reutemann et al. (2023), self‐maters show high morphological variation within populations, compared to outcrossers, while high morphological variation within populations leads to low differentiation among populations (Reutemann et al., 2023). This might be attributed to low meiotic recombination in *Paspalum* selfers since they are believed to be apomictic, a trait of great importance in breeding of clonal seeds (Ortiz et al., 2013). In *Paspalum*, an unreduced embryo sac develops from nucellar cells, while the male germ line often shows irregular meiosis (multivalents, asynapsis, chromosome bridges, and micronuclei) and defective pollen mitosis (irregular cytokinesis) (Ortiz et al., 2013). Previous work by this team suggests that apomixis might be linked to deregulated expression of *MAPK3* genes and homologs of *LORELEI* (Ortiz et al., 2013). *Mitogen‐Activated Protein Kinase* (*MAPK*) genes have been associated with meiotic and mitotic cytokinesis (De Storme & Geelen, 2013; Lei et al., 2020), while *LORELEI* (*LRE*) codes for a pollen tube receptor localized at the cell wall in synergid cells of the ovule (Hafidh & Honys, 2021). In Argentina, apomixis is a desirable trait because it allows the distribution of clonal seed (Ortiz et al., 2013).

The most economically damaging weed of tropical and subtropical countries is *Cyperus rotundus*, a former Ayurvedic medicinal plant from India (Peerzada, 2017). *Cyperus rotundus* is considered the main weed of maize (*Zea mays* L.), rice (*Oryza sativa* L.), sugarcane (*Saccharum officinarum* L.), and many vegetables (Peerzada, 2017). Moreover, it has the potential to suppress the growth of most crops through strong allelopathic competition (Peerzada, 2017). The work of André Luís Laforga Vanzela at the State University of Londrina in Brazil (Roca et al., 2023) (see Table 1) and Chaves et al. (2023) at the Federal University of Lavras in Minas Gerais, also in Brazil, has shown that the meiosis of Cyperids points to the presence of holocentric chromosomes. In these chromosomes, multiple centromeric units are distributed along the surface of metaphase chromosomes, extending from one telomere to the other, and are therefore seen as an unbroken thread on each chromatid (Hofstatter et al., 2022). Holocentromeres may stabilize chromosomal fragments and fusions that cause karyotype rearrangements and allow speedy speciation (Hofstatter et al., 2022). Holocentric chromosomes may also show high telomerase activity during meiosis which allows for de novo synthesis of broken telomeres (Jankowska et al., 2015). Therefore, in Cyperids, dysploidy, together with fissions and fusions, is common during meiosis; however, pollen viability is unusually high because segregation errors are rectified by the end of meiosis (Chaves et al., 2023). High temperatures caused by climate change may reduce herbicide effectiveness against *Cyperus rotundus*; however, a combination of intercropping, crop rotation, intercropping, the use of cover crops such as *Mucuna aterrima*, and the use of advanced pre‐emergence and post‐emergence herbicides may allow for some degree of control (Peerzada, 2017).

## 4 SEED DEVELOPMENT

Another avenue for plant reproduction research, both basic and applied, is seed development. In the case of *A. thaliana*, the work of Zumajo‐Cardona et al. from Colombia (see Table 1) approaches a post‐zygotic developmental barrier, called the endosperm triploid block (2 maternal: 1 paternal), which results in the non‐viability of crosses between lines of different ploidy to generate viable seeds (Zumajo‐Cardona et al., 2023). The author's work and that of her colleagues have shown that a mutant allele for the transcription factor for flavonoid synthesis, *TRANSPARENT TESTA 8* (*At4g09820*), rescues this block in F1 seeds. Also, this work assessed whether flavonoid biosynthetic genes at the testa allow for maternal‐zygotic crosstalk and somehow control the pace of cellularization of the endosperm (Zumajo‐Cardona et al., 2023). The group of Pabón‐Mora et al. at the Institute of Biology of the University of Antioquia in Colombia has also made several notable contributions to understanding sexual plant reproduction, such as the functional characterization of the bHLH genes *ALCATRAZ* and *SPATULA*, which determine petal expansion and repress fruit lignification in *Solanaceae*, in collaboration with C. Ferrándiz at the Polytechnic University of Valencia in Spain (Ortiz‐Ramírez et al., 2019), and the characterization of orchid homologs of flowering regulator *FLOWERING LOCUS T* in species of horticultural value (Ospina‐Zapata et al., 2020).

Regarding seed development, work by the team of Stewart Gillmor at the Mexican National Laboratory of Genomics for Biodiversity (LANGEBIO) (see Table 1) has shown that in Arabidopsis Columbia x Landsberg hybrid zygotes, the maternal and paternal genomes are transcribed unevenly, probably caused by differences in CpG methylation that affect the expression of key developmental genes such as *MONOPTEROS* (*At1g19850*, for vascular development in embryos) and *GAMETE EXPRESSED PROTEIN1* (*At5g55490*, for nuclear fusion during double fertilization) (Alaniz‐Fabián et al., 2022). Collaborative work of Dr. Gillmor with the team of Daoquan Xiang at the Aquatic and Crop Resource Development Center in Saskatoon, Canada, and several researchers from the Huazhong Agricultural University in Wuhan, China, suggest that in reciprocal crosses of tetraploid and hexaploid wheat lines, there is extensive transcriptional change in F1 hybrid embryos. For instance, alternative splicing, protein processing, and chromatin remodeling show variation in pentaploids from a hexaploid (6n) female parent, whereas protein modification and transport show changes in pentaploids from a tetraploid (4n) female parent. These results were interpreted as suggesting ploidy‐dependent expression of imprinted genes (Jia et al., 2022). This type of work is very relevant since for the last 50 years the International Maize and Wheat Improvement Center (CIMMYT) has performed wheat breeding at the Norman E. Borlaugh research station near Ciudad Obregón, in Sonora State, Mexico, and released over 30 different varieties in China, Egypt, Pakistan, Australia, and Ethiopia (Mondal et al., 2020). Moreover, genetically modified wheat that is drought‐tolerant has been developed in Argentina, and its cultivation may become widespread in South America (Roca et al., 2023), so research into the biology of wheat seed development and ploidy may be of even more value in the future.

Seed germination is difficult when the environment is saline (Palomar et al., 2021). The work of Alejandra A. Covarrubias at UNAM university in Mexico (see Table 1) has shown that in Arabidopsis, the RNA‐directed DNA methylation pathway represses the activation of transposable elements during salinity stress (Palomar et al., 2021) and that mutations in the endonuclease ARGONAUTE 4 (AGO4, At2g27040) reduce the ability to cope with salinity (Palomar et al., 2021). Moreover, the localization of AGO4 within the embryo is dynamic, and the AGO4‐GFP protein mostly relocalizes to the vascular tissues from cotyledons during salinity stress (100 mM NaCl) (Palomar et al., 2021). Soil salinity is a threat to global food security and environmental sustainability, and strategies of adaptation are believed to be the best way to deal with it in the face of climate change (Mukhopadhyay et al., 2021).

In maize, the work of Dinkova et al. at UNAM university in Mexico (see Table 1) in collaboration with Blake C. Meyers at the University of Missouri has shown quite elegantly that for zygotic embryos to proliferate in vitro there needs to be strong expression of microRNAs, trans‐acting siRNAs, and heterochromatic siRNAs as induced by treatment with auxins, especially in samples collected 15 days after pollination (Juárez‐González et al., 2019). These transcriptional patterns may be linked to the control of *Copia*, *Gypsy*, and other transposable elements (Juárez‐González et al., 2019). A better understanding of transcriptional patterns may allow for better selection of maize calli with high embryogenic potential (Juárez‐González et al., 2019). Maize genetic transformation is performed in calli (Sun et al., 2013).

The role of hormonal regulation of female gametogenesis is a key developmental process that has been studied in Arabidopsis by the team of Pagnussat et al. at the Mar del Plata University in Argentina (see Table 1) in collaboration with Jana Oletskava at the Czech Academy of Sciences (Bellido et al., 2022). The collaboration found that genes coding for mitochondrial adrenodoxins 1 and 2 and adrenodoxin receptor (*ADX1*, *At4g05450*; *ADX2*, *At4g21090*; and *ADXR*, *At4g32360*) control the fusion of the polar nuclei and placement of the synergids within the ovule. After pollination, embryo development is compromised, probably because of defects in the transfer of electrons within the mitochondrial P450 pathway (Bellido et al., 2022). In the mutants, pollen tube attraction is defective as well (Bellido et al., 2022). Animal ADXR proteins also have a role in steroid synthesis, so the team reasoned that exogenous application of the plant brassinosteroid called homocastasterone would reduce developmental seed abortion (Bellido et al., 2022). Subsequent application of homocastasterone may have reduced abortion from about 35%–5% (Bellido et al., 2022). The team thinks that this common steroid molecular pathway between plants and animals might be due to convergent evolution (Bellido et al., 2022). In China, female sterility is used for breeding hybrid rice (Xia et al., 2019), so an agricultural application might be developed for inducing female sterility by *adx1/2* in Argentina, for rice or for soy.

## 5 POLLEN TUBE DEVELOPMENT

The study of plant reproduction could not be completed with a good understanding of pollen tube development. The work of Muschietti and Estevez (see Table 1) at the Institute of Genetics and Molecular Biology of the Argentinian National Scientific and Technical Research Council (INGEBI‐CONICET) in Buenos Aires and the Millennium Institute for Integrative Biology (iBio), in Santiago, Chile, respectively, has shown that in Arabidopsis, proper elongation of tubes requires the activity of the proline‐rich extensin‐like receptor kinases, which belong to the hydroxyproline‐rich glycoprotein (HRGP) superfamily (Borassi et al., 2021). Staining of the cell wall with pontamine fast scarlet 4B (S4B) suggested that the cell wall of mutant pollen tubes germinated in vitro is thicker, whereas staining with 2,7‐dichlorofluorescein diacetate probe showed excessive production of reactive oxygen species, affecting the deposition of pectins and thereby impairing elongation and seed production (Borassi et al., 2021). More recently, this team suggested that the HRGP superfamily glycosylates leucine‐rich repeat extensins (LRXs) (Sede et al., 2022). The process involves the genes *PROLYL 4‐HYDROXYLASE ISOFORM 4/6* (*P4H4*/*6*, loci unreported), as deduced by defects in germination and elongation observed in the Arabidopsis T‐DNA double mutants, plus the corresponding complementation assays and subcellular localization studies with P4H4‐YFP lines (Sede et al., 2022). Also, mutant pollen tubes were plasmolyzed after germination with a mannitol solution at 40% v/v and the fluorescence of LRX11‐GFP was compared to the fluorescence pattern of membrane dye propidium iodide (PI). In the wild type, most of the LRX11‐GFP signal colocalized with PI at the apoplast of the pollen tip (80%), but very little LRX11‐GFP did so in the mutant line *p4h4‐1p4h6–1* (20%), which suggests that glycosylation of LRX11 by P4HS may affect the hydroxylation of LRXs in the Golgi apparatus and allow for properly glycosylated LRXs to be ferried into exocytic vesicles and then delivery to the apoplast, where they partake in the crosslink and assembly of the cell wall (Sede et al., 2022). LRXs may be required for polarized cell growth by interacting with their N‐terminal domain with the rapid alkalinization‐factor peptides at the cell wall, where they activate reactive oxygen species and calcium signaling pathways (Sede et al., 2022), and this article may lend strong support to this hypothesis. The study of polarized cell growth in pollen tubes has long been deemed of great agricultural value (Zhang & McCormick, 2008), especially the interplay between climate change, pollen tube development, and yield across crops such as rice, maize, potato, and wheat (Chaturvedi et al., 2021).

In Chile, the team of Simón Ruiz‐Lara (see Table 1) at the Millennium Nucleus for the Development of Super Adaptable Plants (MN‐SAP), in Santiago, and at the University of Talca have characterized in Arabidopsis the role of a C_2_H_2_‐type zinc‐finger transcription factor called AtZAT4 (*At2g45120*), which was suspected to operate similar to DUO POLLEN 1‐ACTIVATED ZINC FINGER 1/2 (Puentes‐Romero et al., 2022). Alexander staining of pollen from the *Atzat4* (+/−) line did not reveal any viability defects; however, in vitro germination showed reduced germination and short tubes when compared with the wild type. Seed set counts and seed viability as measured by staining with tetrazolium suggested that *AtZAT4* might have an additional role in embryo development, but more studies are needed to better assess this hypothesis (Puentes‐Romero et al., 2022). In Brazil, Goldman et al. at the University of São Paulo (see Table 1) used *Nicotiana tabacum* L. cv Petit Havana instead of Arabidopsis to characterize the role of a putative pectin acetylesterase (PAE) gene called *NtPAE1* during pollen tube elongation and seed formation (Lubini et al., 2023). To do this in a crop such as Nicotiana, stigmas/styles and ovaries were collected at each stage of flower development (12 in total) for mRNA extraction and RT‐qPCR, followed by in situ hybridization, cloning, and plant transformation with *Agrobacterium tumefaciens* strain C58C1RifR (pGV2260) (Lubini et al., 2023). The role of *NtPAE1* in the wild type and the T‐DNA mutant (Ri16.2) was fully determined by pollen tube growth analyses on stigmas and styles collected 7 h after pollination (Lubini et al., 2023). Pollen tubes from the mutant were not able to elongate significantly in the wild type, and mutant plants were sterile (Lubini et al., 2023). Pollen acetylesterases are important in crops including olives (*Olea europaea*) (Lubini et al., 2023), so this line of research might have a direct agricultural impact in South America in areas that grow olives, such as Chile.

## 6 CONCLUSIONS AND OUTLOOK

Taken together, these reports are evidence that in Latin America, there is a roster of young scientists doing high‐quality research in plant reproduction. However, the teams require more government support for financial stability, to overcome institutional underfunding and overbearing bureaucracy and to be better prepared to face the challenge of developing socially relevant projects (Miranda‐Nieto et al., 2022). In developing countries, translational research from model plants into local crops should be considered a top priority and lead to the development and transfer of high‐yielding crop varieties to farmers in the face of loss of arable land and the effects of climate change (Ronald, 2014). Well‐funded, multinational, and long‐term partnerships to support plant breeding and sustainable agriculture are vital (Ronald, 2014). Thus, local plant reproduction groups must work together and create umbrella consortia, but the actual strategy to do so is not clear.

Latin America features clusters of high‐quality research in plant reproduction centered on Mexico, Brazil, Argentina, Chile, and Colombia, often in collaboration with teams abroad. Continuous cooperation with leading centers in Europe and North America will be necessary to maintain the exchange of students and faculty due to the differences in national incomes and relative expenditures in research and development. For instance, Mexico spends 0.29% of its GDP on science when compared with 2.71% in Germany (https://data.oecd.org/rd/gross‐domestic‐spending‐on‐r‐d.htm).

Plant reproduction research into native crops must be expanded to guarantee food security well into the future, especially in terms of fair and open seed management and distribution among farmers (García‐López et al., 2019). The study of agricultural biodiversity may also pave the way for crops with new and exciting traits, as seen with native *Vanilla* species (Watteyn et al., 2023). Finally, plant and agricultural research in Latin America may have a positive multiplying role in growth of purchasing power, nourishment of rural children, and social stability (Pawlak & Kołodziejczak, 2020). Therefore, research into plant reproduction needs to be encouraged not only for scientific advancement but also for creating more resilient societies.

**FIGURE 1:**
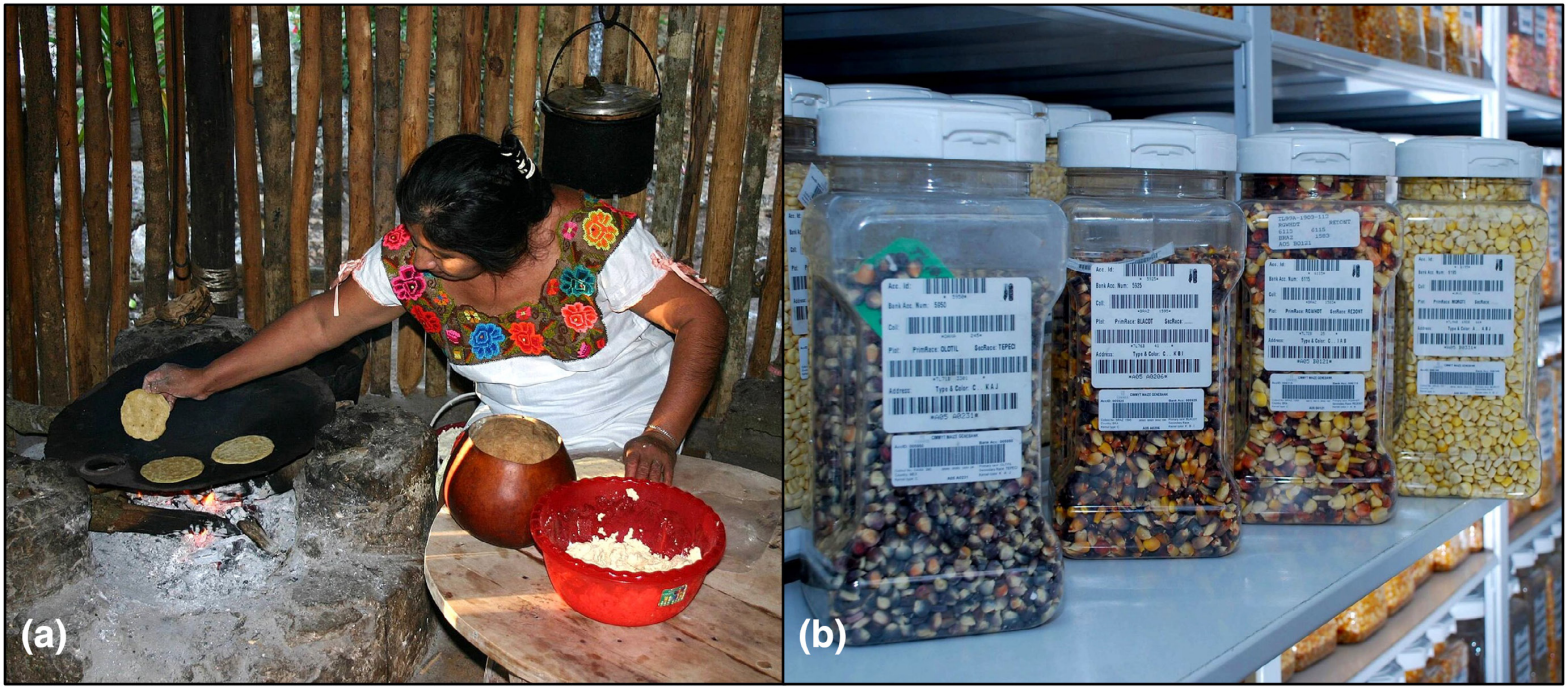
Latin American agricultural diversity is the result of ancestral plant breeding at rural communities (a) and it can be collected and characterized (b) to breed crops that enhance food security and facilitate the adaptation to anthropogenic climate change. a, Picture taken at Yucatán Mexico. b, Picture taken at the seed vault of CIMMYT in Texcoco, Mexico. Credits: (a) *AlaskanDude* via FLICKR and Wikimedia. (b) *CIMMYT* via FLICKR. Files are licensed under a Creative Commons Attribution 2.0 Generic license.

An overlooked aspect of plant reproduction and plant breeding in Latin America is that much of it was carried in the past before the advent of modern science and that much of the preexisting agricultural diversity still exists in rural communities (Ureta et al., 2020) (Figure 1a). Seeds are the result of millennia of multigenerational and collaborative labor and knowledge‐making (Fullilove, 2024). Thus, to protect present and future community plant breeding, NGOs and universities could devise cost‐effective ways to safeguard landrace seeds within communities, while providing much needed extension services (Ureta et al., 2020), such as the Red de Semillas Libres de Colombia (RSLC; Free Seed Network of Colombia) (García‐López et al., 2019).

Moreover, the world already benefits from plant breeding managed in Latin America. For instance, the International Maize and Wheat Improvement Center (CIMMYT) whose headquarters are in Texcoco (Mexico State) (Figure 1b) has quickly developed a maize breeding pipeline for Kenya, Uganda, and Ethiopia (Eastern Africa Product Profile 1a, EA‐PP1a) that is tolerant to Maize Lethal Necrosis and drought conditions, but also shows genetic gain yields of 2.46% per year (64 kg per hectare per year) (Prasanna et al., 2022). This was accomplished together with the Nairobi and Addis Ababa CIMMYT branch offices with help from the Ugandan government. Another pipeline was also developed for Bangladesh, India, Nepal, and Pakistan in cooperation with local governments. These are drought and heat tolerant lines (South Asia Heat and Drought Stress Tolerance, SADHT lines) that reach yields of 71 kg per hectare per year and show genetic gain yields from 0.24% (high Vapor Pressure Deficit, VPD) to 2.02% (low VPD) (Prasanna et al., 2022). Perhaps accelerated introgression of new traits could be attempted with maize homologs for regulators of meiotic crossover formation such as *FANCM* (Crismani et al., 2012).

In Palmira, Colombia, the International Center for Tropical Agriculture (CIAT) runs the Cassava breeding program (Ospina‐Zapata et al., 2020). Cassava (*Manihot esculenta* Crantz) is considered one of the most important crops in the world, and its successful cultivation is instrumental for keeping food security and income generation for nearly 600 million people (Ospina‐Zapata et al., 2020). The roots are the main product and represent a major source of carbohydrates in the tropics where drought and poor soils restrict the cultivation of any other crop (Ospina‐Zapata et al., 2020). Moreover, cassava leaves are an important source of essential amino acids, vitamins, and minerals both for human consumption and as animal feed across Vietnam, Indonesia, Nigeria, and Brazil (Ospina‐Zapata et al., 2020). Cassava tissues contain low levels of cyanide; thus, they have to be carefully washed. Recently, CIAT has released two lines (VEN77 and PAN51) with low cyanide and β‐carotenoid content that may be used as parental lines for breeding purposes (Ospina‐Zapata et al., 2020), potentially contributing to raising living standards across the developing world. Meiosis in cassava species and hybrids is irregular and poorly understood (Nassar et al., 1995). This may be a niche full of opportunities in the future.

A third route for fostering investment in plant research under chronic low investment in science is to tackle government regulatory red tape related to biotechnology and genome editing, as suggested by Roca et al. (2023). This is a controversial topic, but it is worth noting that climate change may challenge the natural capacity of crops to adapt to rising temperatures, salinity, drought, pests, diseases, and flooding (Raza et al., 2019; Roca et al., 2023). We may assume that deregulation may allow for the quick transfer of advanced technologies from academia (via spinoffs) to the marketplace or to rural communities.

A fourth route is to lobby for better integration of environmental policies with agriculture. This is difficult and tricky in the current context of ideological conflict and societal polarization, as seen recently in Brazil with the Jair Bolsonaro administration, in which a far right government aggressively pushed for deforestation and mining while fiercely attacking researchers (Escobar, 2020). In such dangerous circumstances, decentralized citizen engagement should be prioritized.

In conclusion, Latin American research on plant reproduction and plant breeding operates through successful teams at leading academic institutions, but its societal standing and economic impact would be greatly enhanced by partnering with NGOs that engage with rural communities, by close cooperation with leading plant breeding institutions and by active lobbying on behalf of policies for the adoption of biotechnology and for environmental protection. These are not exclusive efforts, and their success may allow to overcome perpetual underfunding and low interest in science at a time in which climate change requires agricultural adaptation to survive.

## CONFLICT OF INTEREST STATEMENT

The authors declare they do not have any financial or personal relationships with other people or organizations that could inappropriately influence his work such as employment, consultancies, stock ownership, honoraria, paid expert testimony, patent applications/registrations, or grants.

## DECLARATION OF GENERATIVE AI IN SCIENTIFIC WRITING

The authors declare that AI tools were not used during the writing process and that the authorship and all responsibility therein are entirely human.

## SUBMISSION DECLARATION AND VERIFICATION

The authors declare that this manuscript has not been published previously and that it is not under consideration for publication elsewhere, and that, if accepted, it will not be published elsewhere in the same form, in English or in any other language, including electronically without the written consent of the copyright holder.

## SOCIETAL IMPACT STATEMENT

Latin America is home to 700 million people. Latin America is also the origin center of key crops such as maize, potatoes, and tomato. Thus, research into plant sexual reproduction in model organisms and local crops may help elucidate processes related to seed germination, tolerance to salinity, pollen tube elongation, and meiotic recombination in the context of climate change. Support for local research groups and the establishment of collaboration links are necessary in the face of low investment in science across the continent.

## Data Availability

Data sharing not applicable ‐ no new data generated.
